# Case of the Paroxysm of Gout Removed by the Tincture of Colchicum

**Published:** 1814-10

**Authors:** John Want

**Affiliations:** Surgeon to the Northern Dispensary. North Crescent


*For the Medical and Physical Journal.*

### *Case of the Paroxysm of Gout removed by the Tincture of Colchicum; by* Mr. Want.

IT was observed by Dr. Sutton, in his letter on my mode of preparing the Eau Medicinale, that the colchicum, which I consider to be the basis of the remedy, produced its beneficial effects by its purgative operation; and distinctly asserts that even common purgatives are capable of removing the disease with equal facility. For the complete refutation of both these assertions, I may refer to our last Number, where the subject is considered at length; but a case of gout cured by colchicum has since occurred to me, which will, I think, be admitted in proof that purging is not essential to the success of this medicine. It will be observed that in the subject of this case the paroxysm was twice removed,—the first time by a full dose of the remedy, which occasioned, as is most common, a strong action on the bowels; but on the second attack, being attentive to avoid the excitement of purging, the disease was as well cured without it.

James Beddell, residing No. 18, Grenville-street, Somers Town, has been severely afflicted with gout for the period of fourteen years. The paroxysms attack him three or four times a-year, mostly lasting about two months, but latterly much much longer. When he applied to me about six weeks ago, he had been confined to the room about five months. A dram of the dried colchicum only was ordered, but, from some mistake in the gentleman who was to superintend its effects, a second was taken, after an interval of eight hours. The symptoms were nearly removed before the second dose was taken. The man was purged and vomited during the space of four days, with loss of appetite, and intense head-ach. Two days previous to the drawing up of this case, he applied to me for more medicine. The disease had returned in the left knee, thick part of the thigh, and calf of the leg, with swelling of all these parts: the fingers were also affected. Undismayed by the severity of operation he had endured, he requested a repetition of the medicine. Half a dram of fresh colchicum was given to him at ten o'clock in the evening, but the pain continued unabated till night, and at eight next morning he had *one motion without relief*. He took another half dram, and in two hours more began to mend. At half-past one, the time of noting this account, he could move himself more freely, though still unable to walk without a crutch; was able to raise his leg, and keep it, unsupported, in a position nearly horizontal; no more evacuation from the bowels had been experienced. The swelling has left the finger, leg, and thigh, and has almost disappeared in the knee.

The pain felt was described as a gnawing sensation, with no feeling of heat or cold, though in general, in former attacks, the parts affected were very hot, and the urine was scalding. The sensible effects of the remedy, previous to the relief afforded, on this occasion, were not discoverable, if we except the one evacuation; and that this was not the cause of benefit is obvious, from the fact that the pain did not diminish until two hours after, when the second dose had been taken.

Whitehair, a beadle of St. Pancras, obtained from the preceding patient a dose of the medicine. He had been hobbling about Somers Town for several months, and, after taking it in the evening, was in a few hours completely relieved, and able to walk the following day free from pain. He had four evacuations from the bowels, which commenced about five o'clock in the morning; but he assures me the pain was essentially relieved before that time. I should not consider a case of this kind to be conclusive, because I have certainly seen the same effect from elaterium. Where this has been the case, I have been accustomed formerly to account for it by the supposition that, although no evacuation from the bowels *preceded* the removal of the pain, yet the *morbi fomes* might be dislodged and evacuated from the stomach and upper intestines, testines, perhaps the only parts capable of being influenced by it, long before it was expelled from the body. In many, if not all the cases where the disease has yielded to the colchicum, and vomiting has been excited, a considerable quantity of very viscid bilious matter has been brought up, with great relief of the symptoms. Emetics are also useful in gout. But the colchicum, though it has generally a purgative and often an emetic property, appears to have some specific operation in the treatment of gout, which it remains for future experiments to develope. It produces sleep in most cases where I have used it in this disease, far more readily than the greatest dose of opium. Whether this is the consequence of its real anodyne powers, or of its specific property in curing those forms of gout to which it is applicable, I shall not enter upon at present.

JOHN WANT,
*Surgeon to the Northern Dispensary.*

*North Crescent, Sept.* 1814.

